# Supplementary material for: Different pruning level effects on flowering period and chlorophyll fluorescence parameters of Loropetalum chinense var. rubrum
Source: PeerJ. 2022 May 10;10:e13406. doi: 10.7717/peerj.13406 (PMC9104088; doi:10.7717/peerj.13406)
Supplement: File S4 [file peerj-10-13406-s004.zip › File4/HZZ.htm]

xml version="1.0" encoding="UTF-8"?


HZZ


IBM SPSS Web Report - HZZ.spv

（系统已禁用控制）

- 打印
- 在简化视图中打开
- 在交互式视图中打开

- 对数

  - 对数
- Oneway

  - Active Dataset
  - Descriptives
  - Test of Homogeneity of Variances
  - ANOVA
  - Robust Tests of Equality of Means
  - Post Hoc Tests

    - Multiple Comparisons
    - Homogeneous Subsets

      - Time
  - Means Plots

    - Time

系统缺省设置
传统
传统备用
Cobalt 备用
灰色
灰色备用


A

|  |  |  |  |  |  |
| --- | --- | --- | --- | --- | --- |
|  | |  | |  | |
|  |  |  |  |  |  |
|  |  |  |  |  |  |
|  |  |  |  |  |  |
|  |  |  |  |  |  |
|  |  |  |  |  |  |

对数

GET  
  FILE='H:\Database\Relationship between pruning intensity and initial bloom time-HZZ.sav'.  
DATASET NAME DATA1 WINDOW=FRONT.  
ONEWAY Time BY VAR00001  
  /STATISTICS DESCRIPTIVES EFFECTS HOMOGENEITY BROWNFORSYTHE WELCH  
  /PLOT MEANS  
  /MISSING ANALYSIS  
  /POSTHOC=DUNCAN LSD ALPHA(0.05).

Oneway

Data[1] H:\Database\Relationship between pruning intensity and initial bloom time-HZZ.sav

Oneway

DescriptivesDescriptives, 表, Time, 1 层、列标题的 2 级和行标题的 2 级, 具有 11 列和 11 行的表

| |  |  | | --- | --- | | Time | Time | | | | | | | | | | | |
|  |  |  |  |  |  |  |  |  |  |  |
| --- | --- | --- | --- | --- | --- | --- | --- | --- | --- | --- |
|  | | N | Mean | Std. Deviation | Std. Error | 95% Confidence Interval for Mean | | Minimum | Maximum | Between- Component Variance |
|  |  |  |  |  |  | Lower Bound | Upper Bound |  |  |  |
| A | | 5 | .0000 | .00000 | .00000 | .0000 | .0000 | .00 | .00 |  |
| B | | 5 | .0000 | .00000 | .00000 | .0000 | .0000 | .00 | .00 |  |
| C | | 5 | 19.8000 | .83666 | .37417 | 18.7611 | 20.8389 | 19.00 | 21.00 |  |
| CK | | 5 | 22.6000 | .54772 | .24495 | 21.9199 | 23.2801 | 22.00 | 23.00 |  |
| Total | | 20 | 10.6000 | 10.93233 | 2.44454 | 5.4835 | 15.7165 | .00 | 23.00 |  |
| Model | Fixed Effects |  |  | .50000 | .11180 | 10.3630 | 10.8370 |  |  |  |
|  | Random Effects |  |  |  | 6.14654 | -8.9610 | 30.1610 |  |  | 151.07000 |
|  |  |  |  |  |  |  |  |  |  |  |

Oneway

Test of Homogeneity of VariancesTest of Homogeneity of Variances, 表, Time, 1 层、列标题的 1 级和行标题的 0 级, 具有 4 列和 4 行的表

| |  |  | | --- | --- | | Time | Time | | | | |
|  |  |  |  |
| --- | --- | --- | --- |
| Levene Statistic | df1 | df2 | Sig. |
| 10.880 | 3 | 16 | .000 |
|  |  |  |  |

Oneway

ANOVAANOVA, 表, Time, 1 层、列标题的 1 级和行标题的 1 级, 具有 6 列和 6 行的表

| |  |  | | --- | --- | | Time | Time | | | | | | |
|  |  |  |  |  |  |
| --- | --- | --- | --- | --- | --- |
|  | Sum of Squares | df | Mean Square | F | Sig. |
| Between Groups | 2266.800 | 3 | 755.600 | 3022.400 | .000 |
| Within Groups | 4.000 | 16 | .250 |  |  |
| Total | 2270.800 | 19 |  |  |  |
|  |  |  |  |  |  |

Oneway

Robust Tests of Equality of MeansbRobust Tests of Equality of Means, 表, Time, 1 层、列标题的 1 级和行标题的 1 级, 具有 5 列和 7 行的表

| |  |  | | --- | --- | | Time | Time | | | | | |
|  |  |  |  |  |
| --- | --- | --- | --- | --- |
|  | Statistica | df1 | df2 | Sig. |
| Welch | . | . | . | . |
| Brown-Forsythe | . | . | . | . |
|  |  |  |  |  |
| --- | --- | --- | --- | --- |
| a. Asymptotically F distributed. | | | | |
| b. Robust tests of equality of means cannot be performed for Time because at least one group has 0 variance. | | | | |
|  |  |  |  |  |

Post Hoc Tests

Multiple ComparisonsMultiple Comparisons, 表, Dependent Variable, Time, 1 层、列标题的 2 级和行标题的 3 级, 具有 8 列和 17 行的表

| |  |  | | --- | --- | | Time | Time | | | | | | | | |
|  |  |  |  |  |  |  |  |
| --- | --- | --- | --- | --- | --- | --- | --- |
|  | (I) VAR00001 | (J) VAR00001 | Mean Difference (I-J) | Std. Error | Sig. | 95% Confidence Interval | |
|  |  |  |  |  |  | Lower Bound | Upper Bound |
| LSD | A | B | .00000 | .31623 | 1.000 | -.6704 | .6704 |
|  |  | C | -19.80000\* | .31623 | .000 | -20.4704 | -19.1296 |
|  |  | CK | -22.60000\* | .31623 | .000 | -23.2704 | -21.9296 |
|  | B | A | .00000 | .31623 | 1.000 | -.6704 | .6704 |
|  |  | C | -19.80000\* | .31623 | .000 | -20.4704 | -19.1296 |
|  |  | CK | -22.60000\* | .31623 | .000 | -23.2704 | -21.9296 |
|  | C | A | 19.80000\* | .31623 | .000 | 19.1296 | 20.4704 |
|  |  | B | 19.80000\* | .31623 | .000 | 19.1296 | 20.4704 |
|  |  | CK | -2.80000\* | .31623 | .000 | -3.4704 | -2.1296 |
|  | CK | A | 22.60000\* | .31623 | .000 | 21.9296 | 23.2704 |
|  |  | B | 22.60000\* | .31623 | .000 | 21.9296 | 23.2704 |
|  |  | C | 2.80000\* | .31623 | .000 | 2.1296 | 3.4704 |
|  |  |  |  |  |  |  |  |
| --- | --- | --- | --- | --- | --- | --- | --- |
| \*. The mean difference is significant at the 0.05 level. | | | | | | | |
|  |  |  |  |  |  |  |  |

Homogeneous Subsets

TimeTime, 表, 列标题的 2 级和行标题的 2 级, 具有 6 列和 10 行的表

|  |  |  |  |  |  |
| --- | --- | --- | --- | --- | --- |
|  | VAR00001 | N | Subset for alpha = 0.05 | | |
|  |  |  | 1 | 2 | 3 |
| Duncana | A | 5 | .0000 |  |  |
|  | B | 5 | .0000 |  |  |
|  | C | 5 |  | 19.8000 |  |
|  | CK | 5 |  |  | 22.6000 |
|  | Sig. |  | 1.000 | 1.000 | 1.000 |
|  |  |  |  |  |  |
| --- | --- | --- | --- | --- | --- |
| Means for groups in homogeneous subsets are displayed. | | | | | |
| a. Uses Harmonic Mean Sample Size = 5.000. | | | | | |
|  |  |  |  |  |  |

Means Plots

IBM SPSS Web Report

X

IBM SPSS Web 报告是一种可在浏览器中打开的交互式报告，它包含由 IBM SPSS Statistics 生成的图表、表格及其他输出。因为此文档是单个文件，所以可将其放置在任何网站（无需特定服务器或安装）、公布到共享文件服务器上、复制到便携文件介质上或者通过电子邮件进行分发。Web 报告是可在所有最常用的浏览器的最新版本上打开的 HTML5 文件。

系统已禁用 Web 报告控制。您使用的浏览器禁用了 JavaScript，或者系统以安全措施的方式禁用了 JavaScript。没有 JavaScript，报告会包含所有相同的图表、表格和其他对象作为全功能版本，但交互式功能不可用。请检查浏览器的设置以打开 JavaScript 或者尝试在其他浏览器中打开报告。对于图形输入板或智能手机，您可能需要安装 HTML 查看器应用程序。

### 控件和选项

:   显示或隐藏编辑工具栏。
:   显示帮助。
:   显示“工具”菜单。

### 导航控件

:   打开或关闭导航树
:   在文档中显示上一个对象（将跳过非公开对象）。
:   在文档中显示下一个对象（将跳过非公开项）。

### 编辑工具栏

您可以使用编辑工具栏来修改表格外观，包括更改字体属性、背景颜色、和小数位数。您还可以将“表格外观”应用到整个表格并变换行和列。您无法更改表格中的数据值，也无法编辑除表格外的对象。

### “工具”菜单

**打印.** 打印 Web 报告的全部内容。

**在简化视图中打开.** 在简化视图中显示 Web 报告的全部内容。会显示全部内容，而不只是显示选中的输出对象。您可以在多维表格中更改层，但您无法对表格作任何其他更改。
