# Supplementary material for: Different pruning level effects on flowering period and chlorophyll fluorescence parameters of Loropetalum chinense var. rubrum
Source: PeerJ. 2022 May 10;10:e13406. doi: 10.7717/peerj.13406 (PMC9104088; doi:10.7717/peerj.13406)
Supplement: File S5 [file peerj-10-13406-s005.pdf]

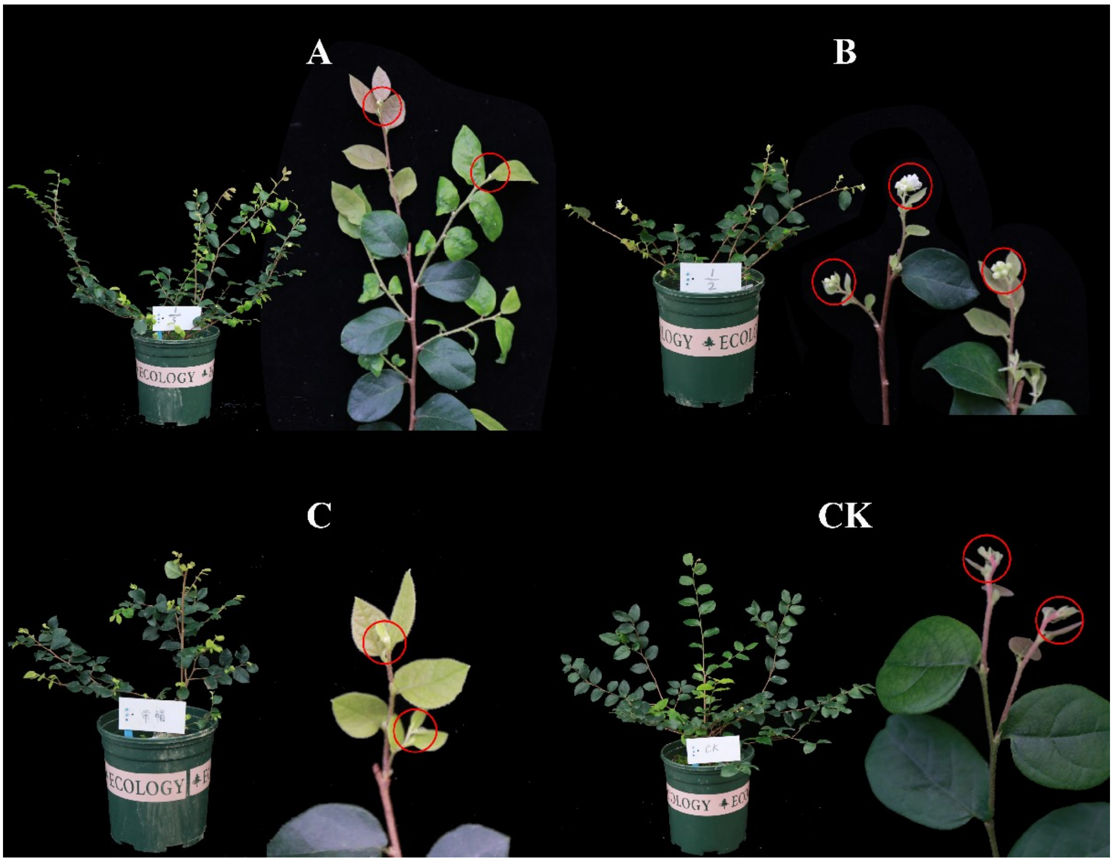

Figure 1. Comparison photos of the treatment group and the control group at the first flowering stage of "Xiangnong Xiangyun"

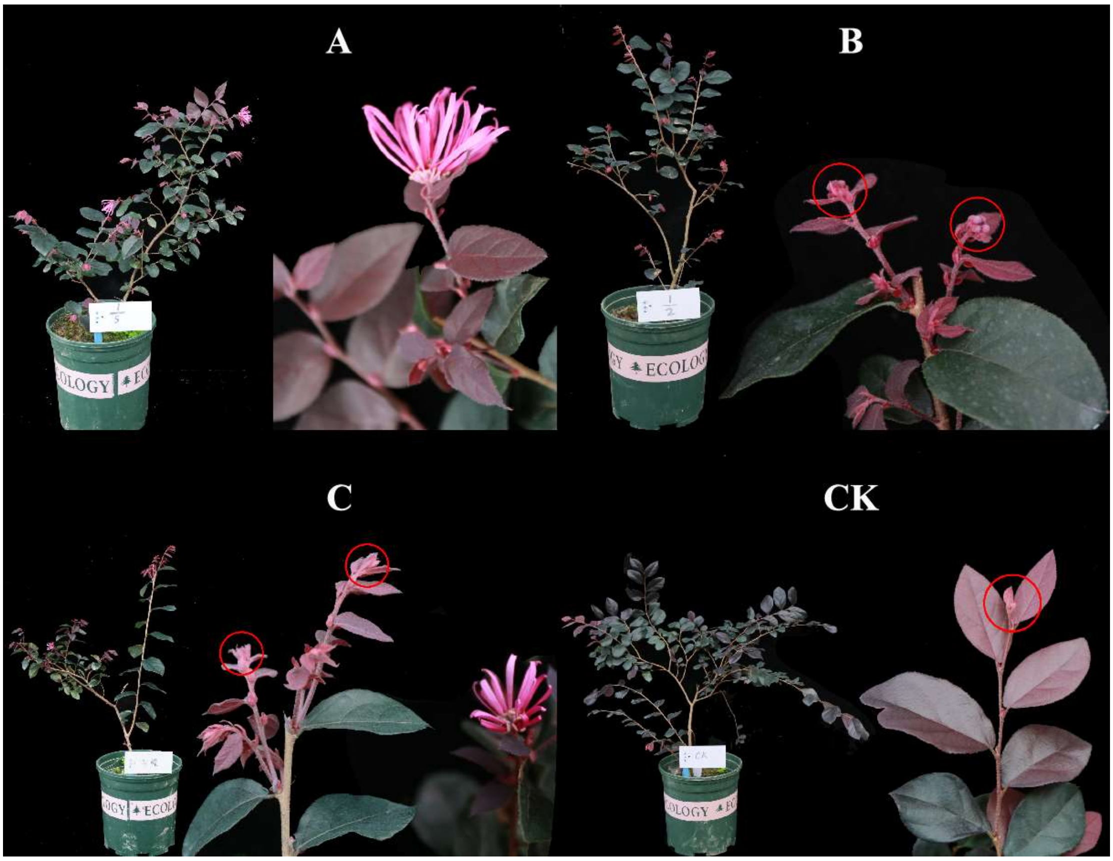

Figure 2. Comparison photos of the treatment group and the control group at the first flowering stage of "Da Yehong"

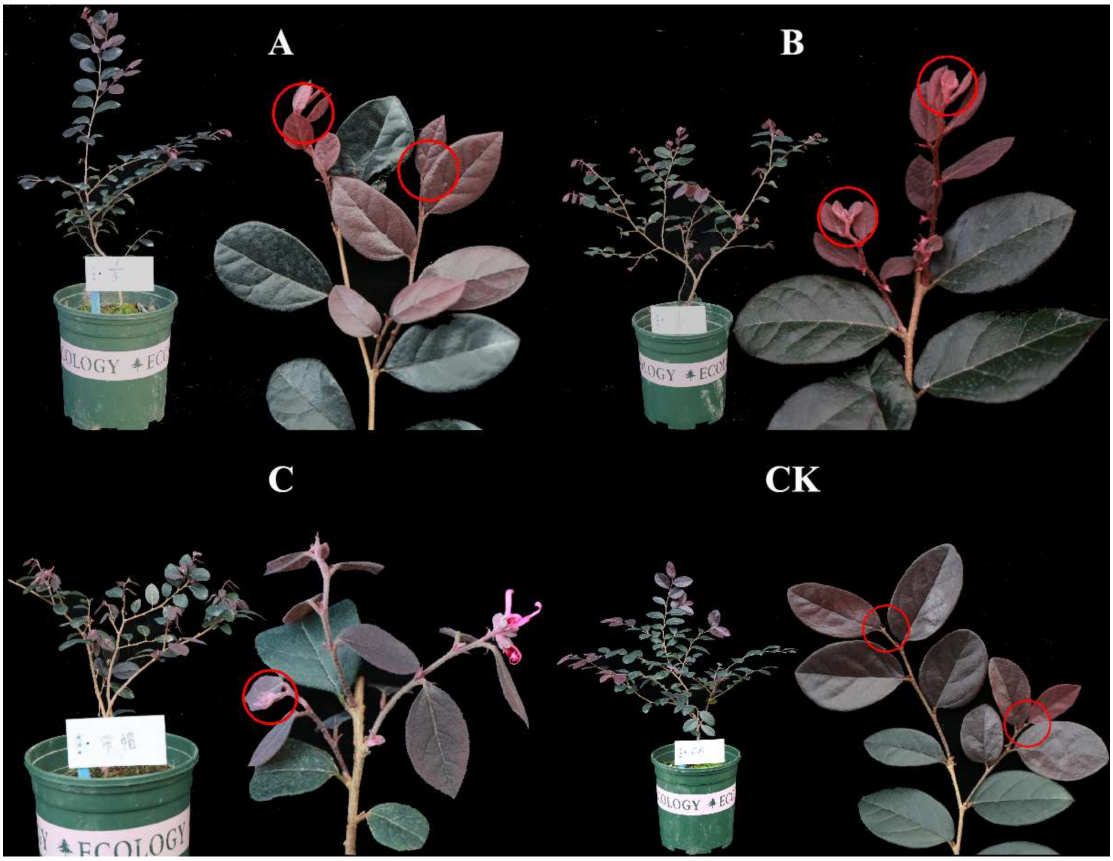

Figure 3. Comparison photos of the treatment group and the control group at the first flowering stage of "Hei Zhenzhu"
