# Supplementary material for: Different pruning level effects on flowering period and chlorophyll fluorescence parameters of Loropetalum chinense var. rubrum
Source: PeerJ. 2022 May 10;10:e13406. doi: 10.7717/peerj.13406 (PMC9104088; doi:10.7717/peerj.13406)
Supplement: File S6 [file peerj-10-13406-s006.pdf]

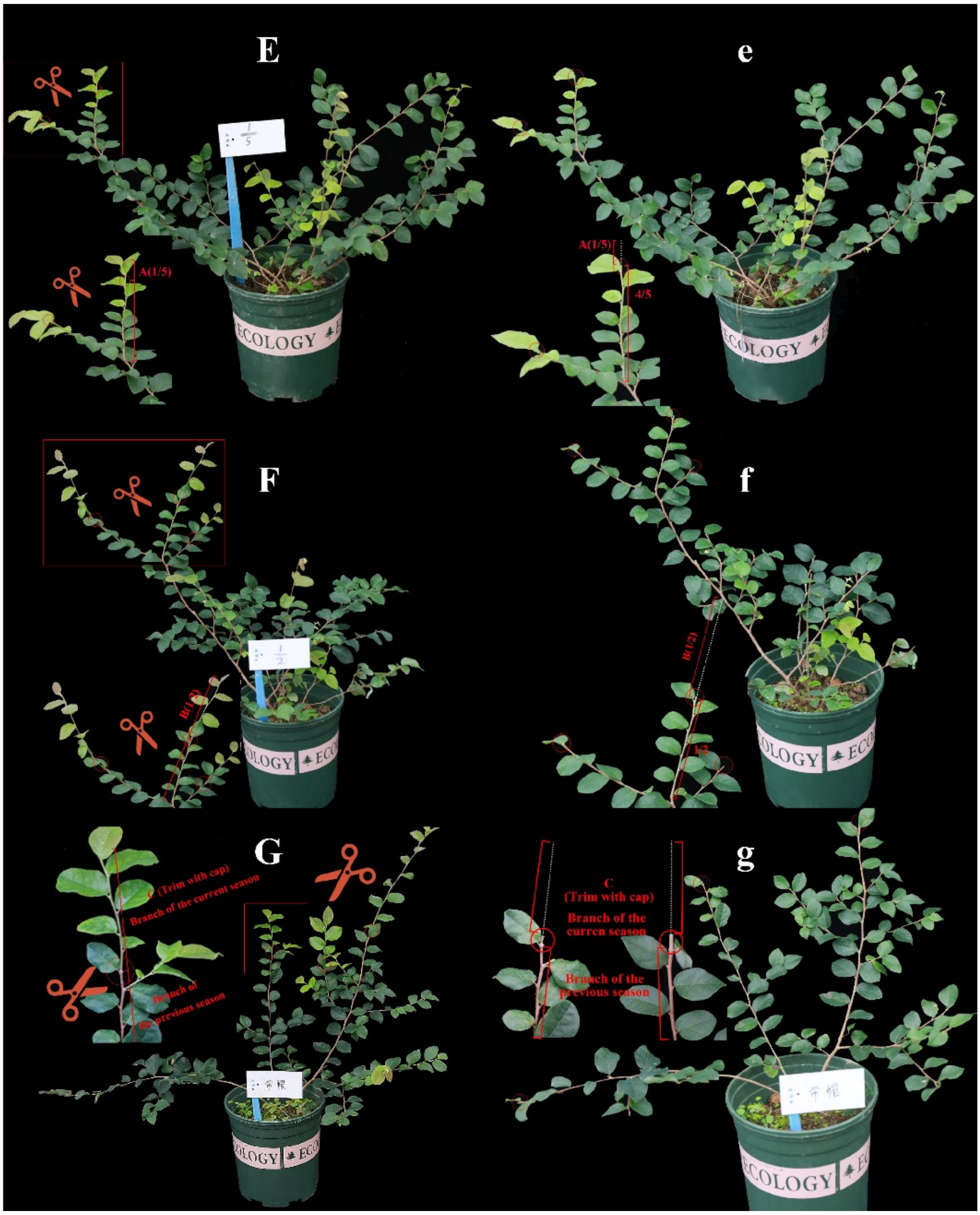

Figure 1. Comparison of "Xiangnong Xiangyun" before and after pruning

Note: Capital letters indicate before pruning; Lowercase letters indicate after pruning; ‘E’ stands for ‘A’ experimental group, ‘F’ stands for ‘B’ experimental group, ‘G’ stands for ‘C’ experimental group.

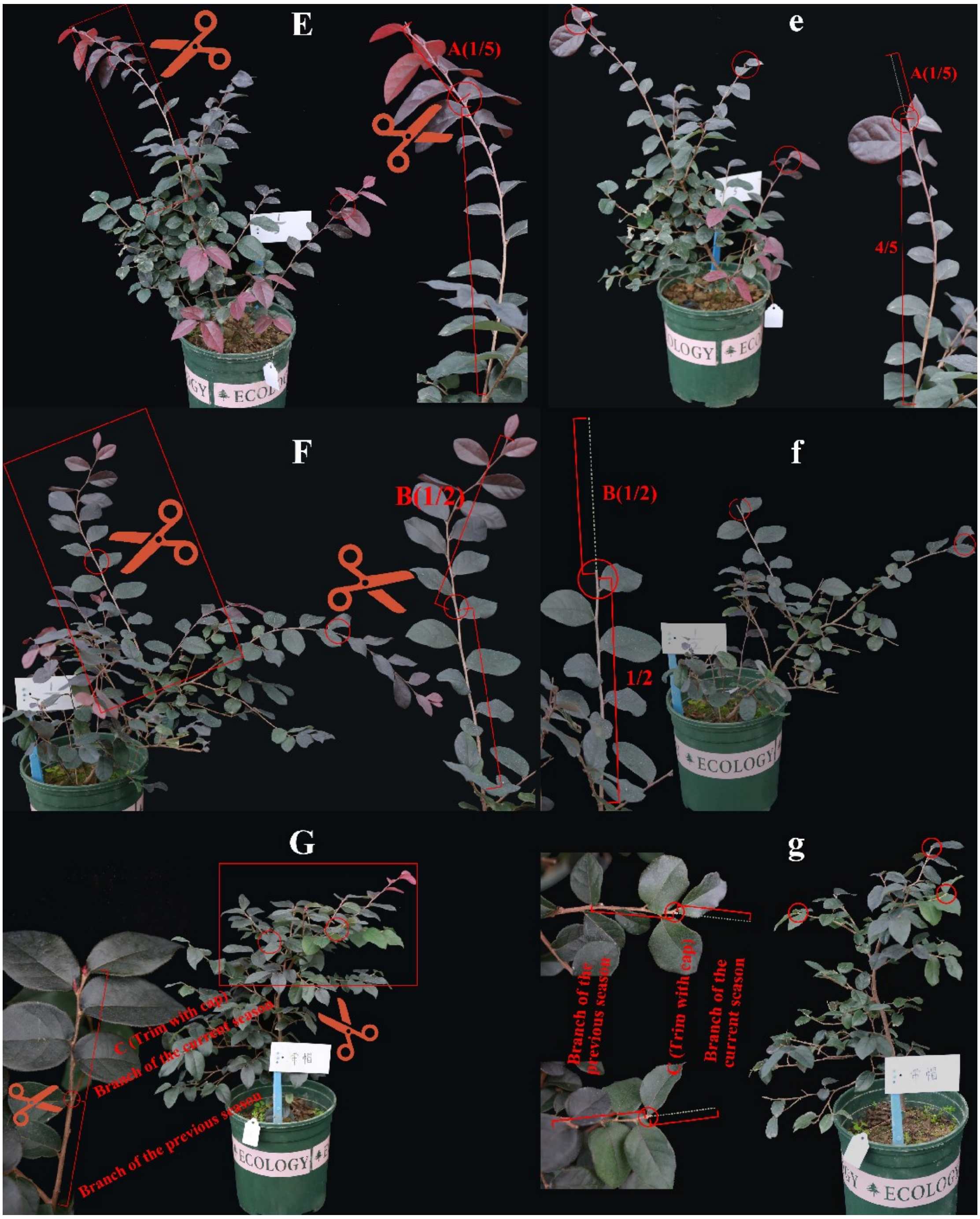

Figure 2. Comparison of "Da Yehong" before and after pruning

Note: Capital letters indicate before pruning; Lowercase letters indicate after pruning; ‘E’ stands for ‘A’ experimental group, ‘F’ stands for ‘B’ experimental group, ‘G’ stands for ‘C’ experimental group.

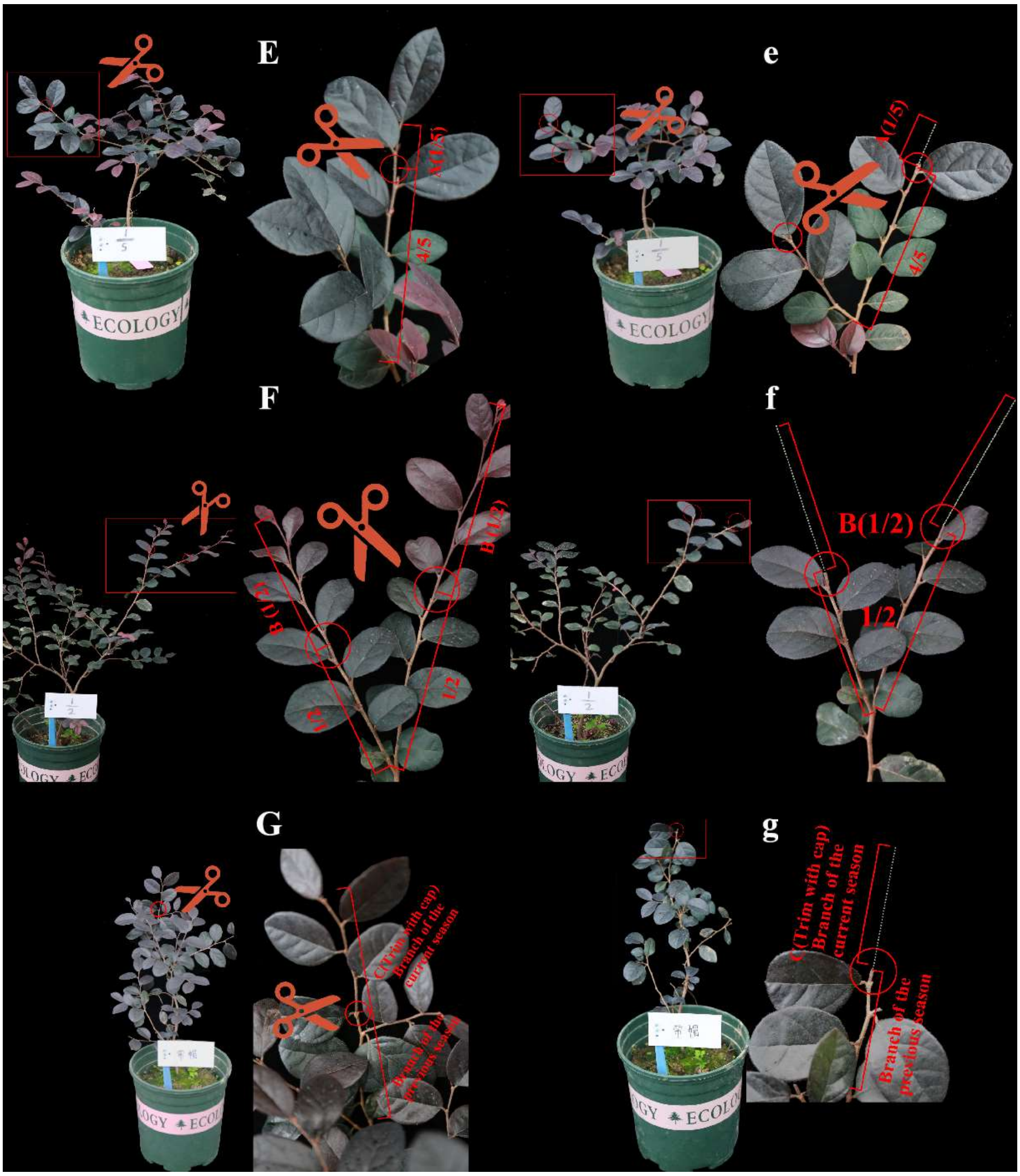

Figure 3. Comparison of "Hei Zhenzhu" before and after pruning

Note: Capital letters indicate before pruning; Lowercase letters indicate after pruning; ‘E’ stands for ‘A’ experimental group, ‘F’ stands for ‘B’ experimental group, ‘G’ stands for ‘C’ experimental group.
